# Hemorrhagic epididymal cyst mimicking testicular torsion: A case report

**DOI:** 10.1016/j.ijscr.2025.111476

**Published:** 2025-06-06

**Authors:** Salim Ouskri, Mohammed Ali Mikou, Idriss Ziani, Imad Boualaoui, Hachem El Sayegh, Yassine Nouini

**Affiliations:** IBN SINA HOSPITAL, Morocco

## Abstract

**Introduction:**

Testicular torsion is a surgical emergency requiring rapid diagnosis and treatment to prevent testicular loss. However, other conditions, such as hemorrhagic epididymal cyst rupture, can mimic its presentation, posing diagnostic challenges.

**Case presentation:**

A 25-year-old male presented with sudden, severe right scrotal pain. Examination revealed a tender superior epididymal mass. Scrotal ultrasound showed homogeneous fluid collection with normal testicular blood flow. Due to severe pain, surgical exploration ruled out torsion and confirmed a hemorrhagic epididymal cyst, which was excised intact.

**Discussion:**

Distinguishing testicular torsion from hemorrhagic epididymal cyst rupture is crucial. Doppler ultrasound remains the primary diagnostic tool, with torsion showing absent blood flow, whereas cysts maintain normal or increased vascularization. In unclear cases, surgical exploration is justified to prevent testicular loss.

**Conclusion:**

Although testicular torsion is the most critical cause of acute scrotal pain, alternative diagnoses, such as hemorrhagic epididymal cyst rupture, should be considered. Prompt diagnosis using clinical evaluation and imaging can prevent unnecessary orchiectomy, with surgical exploration remaining the safest option in uncertain cases.

## Highlights

Introduction

• Testicular torsion is a surgical emergency requiring rapid diagnosis and treatment.
• Prompt differentiation from hemorrhagic epididymal cyst rupture is essential to prevent unnecessary orchiectomy.

Case Presentation

• A 25-year-old male presented with acute scrotal pain; ultrasound revealed normal testicular blood flow and an epididymal fluid collection.
• Surgical exploration ruled out torsion and confirmed a hemorrhagic epididymal cyst.

Discussion

• Doppler ultrasound is crucial for differentiating torsion from cyst rupture; surgical exploration remains necessary when uncertainty persists.

Conclusion

• Prompt imaging and surgical exploration can prevent unnecessary orchiectomy in cases of suspected testicular torsion.

## 1 Introduction

Testicular torsion is a urological emergency that requires prompt diagnosis and surgical intervention to prevent testicular ischemia and subsequent loss of function [2]. It is one of the most common causes of acute scrotal pain in adolescents and young adults [3]. However, a wide range of differential diagnoses can mimic the clinical presentation of testicular torsion, including epididymitis, torsion of the testicular appendage, trauma, and hemorrhagic epididymal cysts [4]. Among these, hemorrhagic epididymal cyst rupture is a rare but significant entity that can present with acute scrotal pain, making its differentiation from testicular torsion critical [5,6].

This case report presents a patient with hemorrhagic epididymal cyst mimicking testicular torsion, emphasizing the importance of imaging and clinical correlation in acute scrotal pain evaluation.

## 2 Case presentation

A 25-year-old male presented to the emergency department with acute-onset, severe right scrotal pain. The pain was sudden and reached maximum intensity immediately. The patient had no significant medical history except for a chronic sensation of discomfort in the right testicle.

On physical examination, the right testicle was normally positioned but extremely tender to palpation. Pain was exacerbated over the epididymis, where a firm, renitent, and highly painful mass was noted at the upper pole.

**Fig. 1:**
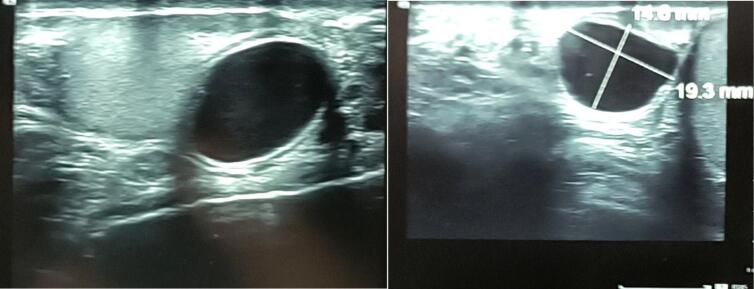
Ultrasound Imaging of a Hemorrhagic Epididymal Cyst.

Emergency scrotal ultrasound revealed a normal-appearing testicle with a well-defined, anechoic, and homogeneous fluid collection measuring approximately **19** **mm × 14** **mm** at the superior pole of the testis, closely associated with the epididymis (Fig. 1).

**Fig. 2:**
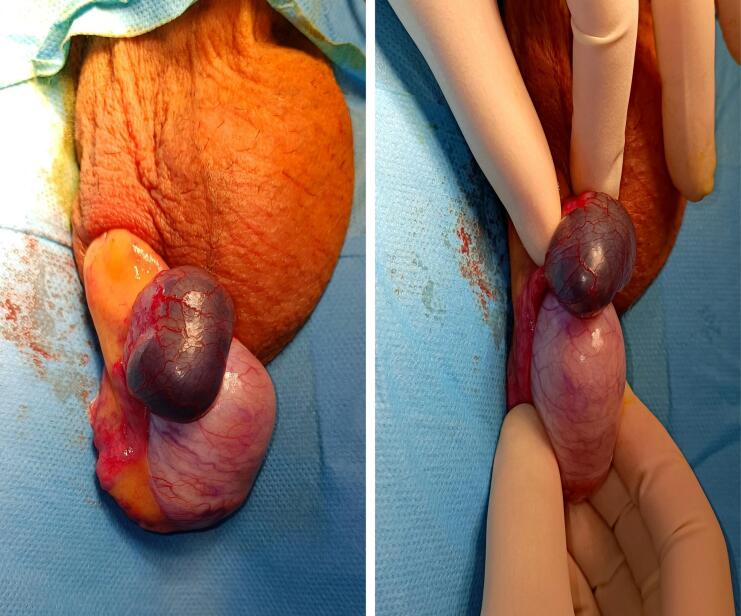
Intraoperative view showing a hemorrhagic epididymal cyst appearing as a dark, engorged mass at the superior pole of the testis.

Due to the severity of pain, the patient was taken to the operating room for an urgent scrotal exploration. Intraoperative findings ruled out testicular torsion, as no twisting of the spermatic cord was observed. Instead, a hemorrhagic epididymal cyst was identified at the superior pole of the epididymis, appearing intact despite intralesional hemorrhage (Fig. 2).

**Fig. 3:**
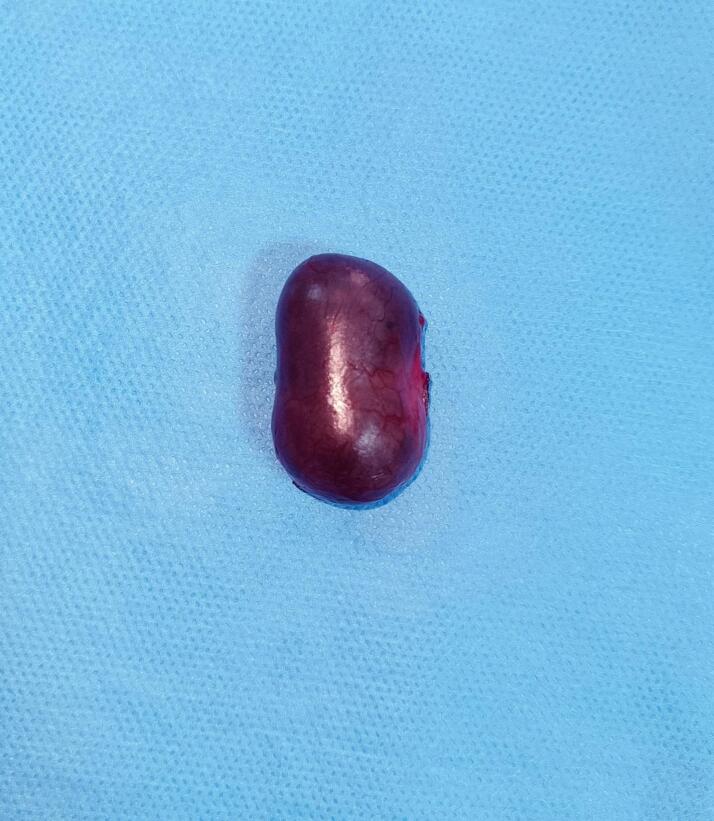
Excised hemorrhagic epididymal cyst, appearing intact after surgical removal.

The cyst was completely excised without rupture (Fig. 3), and the remaining scrotal structures were unremarkable.

## 3 Discussion

Testicular torsion accounts for approximately 25–35 % of acute scrotal pain cases in pediatric and adolescent populations [7]. The estimated incidence is around 5 per 100,000 males under the age of 25 per year, with the highest frequency occurring in neonates and post-pubertal boys [8,9]. Most cases present between the ages of 12 and 18, with a mean age of 14 years [10]. Patients typically present with sudden, severe unilateral scrotal pain, often associated with nausea, vomiting, and an absent cremasteric reflex [11]. As ischemia progresses, scrotal swelling and erythema may develop. Unlike epididymitis, testicular torsion generally lacks fever and urinary symptoms [12].

Hemorrhagic epididymal cyst rupture, although rare, can present similarly to testicular torsion. The sudden onset of scrotal pain in both conditions makes differentiation difficult. However, some key differences exist. Testicular torsion is characterized by the absence of blood flow on Doppler ultrasound, whereas hemorrhagic cysts typically show normal or increased blood flow, along with complex cystic structures containing internal debris [13]. Clinically, patients with hemorrhagic epididymal cysts may report a history of mild, intermittent scrotal discomfort before the acute event, whereas torsion tends to present as a sudden, severe pain without prior symptoms [14].

The diagnostic approach to these conditions primarily relies on imaging, particularly Doppler ultrasound, which remains the gold standard. In testicular torsion, ultrasound findings typically include an enlarged, hypoechoic testis with an absence of blood flow, along with a twisted spermatic cord, often described as the “whirlpool sign” [15]. Conversely, hemorrhagic epididymal cysts appear as heterogeneous cystic structures with internal echoes, sometimes accompanied by surrounding hyperemia, which may mimic epididymo-orchitis rather than torsion [16].

Other imaging modalities, such as MRI, may be useful in equivocal cases, providing additional information on tissue composition and vascular status. Near-infrared spectroscopy is an emerging technique that has shown promise in differentiating between viable and non-viable testes in torsion cases by assessing tissue oxygen saturation [17]. However, its widespread application remains limited.

Ultimately, clinical suspicion remains crucial. Given the devastating consequences of a missed torsion diagnosis, surgical exploration should not be delayed when torsion is suspected. In cases of hemorrhagic epididymal cysts, conservative management with analgesia and follow-up imaging is typically sufficient unless complications such as infection or persistent pain necessitate intervention [18].

## 4 Conclusion

While testicular torsion remains the most critical diagnosis to rule out in cases of acute scrotal pain, clinicians should also maintain a high index of suspicion for alternative diagnoses, such as hemorrhagic epididymal cyst rupture, particularly when ultrasound findings are inconclusive. A thorough clinical evaluation, supported by Doppler ultrasound, is essential to guide decision-making. Given the potentially irreversible consequences of delayed torsion management, any diagnostic uncertainty should still prompt timely surgical exploration to preserve testicular function. Recognizing rare entities like hemorrhagic cyst rupture is key to improving patient outcomes and avoiding unnecessary orchiectomy.

## Author contribution

Salim Ouskri - Urology Resident, IBN SINA HOSPITAL (Corresponding author).

Mohammed Ali Mikou - Urology Resident, IBN SINA HOSPITAL.

Idriss Ziani - Urologist, IBN SINA HOSPITAL.

Imad Boualaoui - Urology Assistant Professor, IBN SINA HOSPITAL.

Hachem El Sayegh - Urology Professor, IBN SINA HOSPITAL.

Yassine Nouini - Urology Professor, IBN SINA HOSPITAL.

## Consent

Written informed consent was obtained from the patient for publication and any accompanying images. A copy of the written consent is available for review by the Editor-in-Chief of this journal on request.

## Ethical approval

Ethical approval is obtained from the ethical comity of the hospital.

## Guarantor

Salim Ouskri.

## Research registration number

N/A

## Methods

This the work has been reported in line with the SCARE criteria.

## Funding

No source of funding.

## Conflict of interest statement

I declare no conflict of interest.
